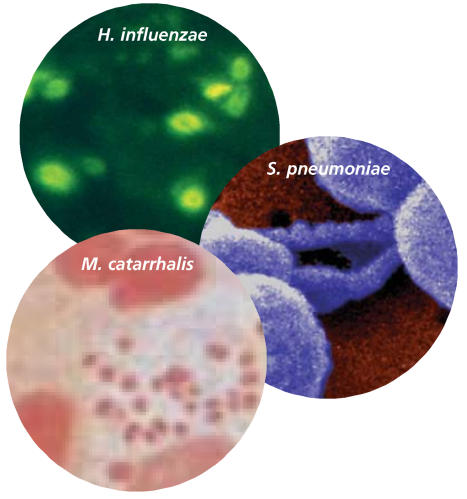# Children’s Health: Do Bacteria Promote Asthma?

**DOI:** 10.1289/ehp.116-a22a

**Published:** 2008-01

**Authors:** Carol Potera

Newborns whose lungs become colonized with certain bacteria are more likely to develop wheezing and childhood asthma than babies not colonized by the bacteria. This connection, reported in the 11 October 2007 issue of the *New England Journal of Medicine*, raises the possibility that controlling bacterial colonization in neonates may lower the prevalence of childhood asthma.

Pediatrician Hans Bisgaard, director of the Danish Pediatric Asthma Center at Copenhagen University Hospital, and colleagues studied 321 asthmatic mothers and their infants for five years. “By including mothers with asthma, we identified a high-risk cohort where we expect to see more children with asthma,” says Bisgaard. A family history of asthma raises the risk for childhood asthma.

At 1 month and 12 months of age, the babies were tested for *Streptococcus pneumoniae*, *Haemophilus influenzae*, *Moraxella catarrhalis*, and *Staphylococcus aureus*. The first three pathogens cause pneumonia, whereas *S. aureus* commonly infects the skin. Parents recorded the children’s respiratory symptoms, such as wheezing and persistent cough, in daily diaries. Physicians examined the children for asthma when they reached 5 years of age.

One-fifth of newborns were colonized with *S. pneumoniae*, *H. influenzae*, *M. catarrhalis*, or some combination thereof, and the presence of 1 or more of these microbes was associated with a 2.4 times greater risk of persistent wheeze and a 3.85 times greater risk of hospitalization for wheezing. Moreover, babies harboring any of these bacteria were about 3 times as likely as children without them to develop asthma by age 5 years. The presence of *S. aureus* was not linked to wheezing or asthma, nor was this bacterium found to colonize the airways of any of the children at age 12 months.

During the first month of life, “children genetically disposed to asthma may be inefficient at clearing pathogenic bacteria from the airways,” proposes Bisgaard as an explanation of his observations. Neonates’ immature immune systems may also be more susceptible to bacterial damage that initiates inflammation leading to asthma, he says. Bisgaard plans to treat a future group of pregnant women and neonates with probiotics (potentially beneficial bacteria) to assess whether such therapy prevents childhood asthma.

“The study raises good questions about immune imbalance associated with asthma,” says Stanley Szefler, head of pediatric clinical pharmacology at the National Jewish Medical and Research Center in Denver. However, he notes, “the positive cultures may be due to an immune imbalance shortly after birth that could lead to bacterial colonization, yet the bacteria [themselves] may not be the direct cause of asthma.” An editorial in the same issue of the *New England Journal of Medicine* makes a similar point—the Danish researchers may have discovered a “new sentinel” for the development of asthma rather than a “causative signal,” writes Erika von Mutius, a professor of pediatrics at the University of Munich.

## Figures and Tables

**Figure:**